# Messenger RNA delivery to mitoribosomes – hints from a bacterial toxin

**DOI:** 10.1111/febs.15342

**Published:** 2020-05-11

**Authors:** Francesco Bruni, Yasmin Proctor‐Kent, Robert N. Lightowlers, Zofia M. Chrzanowska‐Lightowlers

**Affiliations:** ^1^ The Wellcome Centre for Mitochondrial Research Institute of Neuroscience Newcastle University UK; ^2^ Department of Biosciences, Biotechnologies and Biopharmaceutics University of Bari Aldo Moro Italy; ^3^ The Wellcome Centre for Mitochondrial Research Institute for Cell and Molecular Biosciences Newcastle University UK

**Keywords:** LRPPRC/SLIRP, mitochondrial mRNA turnover, mitoribosome, SRL, VapC20

## Abstract

In mammalian mitochondria, messenger RNA is processed and matured from large primary transcripts in structures known as RNA granules. The identity of the factors and process transferring the matured mRNA to the mitoribosome for translation is unclear. Nascent mature transcripts are believed to associate initially with the small mitoribosomal subunit prior to recruitment of the large subunit to form the translationally active monosome. When the small subunit fails to assemble, however, the stability of mt‐mRNA is only marginally affected, and under these conditions, the LRPPRC/SLIRP RNA‐binding complex has been implicated in maintaining mt‐mRNA stability. Here, we exploit the activity of a bacterial ribotoxin, VapC20, to show that in the absence of the large mitoribosomal subunit, mt‐mRNA species are selectively lost. Further, if the small subunit is also depleted, the mt‐mRNA levels are recovered. As a consequence of these data, we suggest a natural pathway for loading processed mt‐mRNA onto the mitoribosome.

## Graphical abstract

The late stages of the life cycle of mitochondrial messenger RNAs (mt‐mRNAs) are still poorly understood; in particular, protection of mt‐RNAs, their recognition by mitochondrial ribosomes and set‐up of translation. Now, Francesco Bruni and colleagues use the bacterial endoribonuclease VapC20 – inducibly expressed in a cell line – as a novel tool to selectively degrade a part of a large subunit of the mitochondrial ribosome. Using this cellular model, they propose that formation of mRNA‐associated ribosomes is needed for mt‐mRNA stability after transfer from the mt‐mRNA‐stabilising LRPPRC/SLIRP complex. For further information, please also see our Commentary by Borowski and Szczesny: https://febs.onlinelibrary.wiley.com/doi/10.1111/febs.15435.

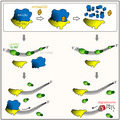

**Abbreviations**
AIF: apoptosis‐inducing factor
EFTu: mitochondrial elongation factor Tu
eIF4E: eukaryotic initiation factor 4E
GDH: glutamate dehydrogenase
HUGO: Human Genome Organization
LRPPRC: leucine rich pentatricopeptide repeat containing
mt: mitochondrial
mt‐LSU: mitochondrial 39S large subunit
mt‐SSU: mitochondrial 28S small subunit
SLIRP: SRA stem‐loop‐interacting RNA‐binding protein
SRL: sarcin:ricin loop
VapC20: virulence‐associated protein C20

## Introduction

The human mitochondrial genome is transcribed from single promoter regions on each of the two strands [1, 2]. The resultant large polycistronic RNA units are processed into individual messenger (mt‐mRNA), ribosomal (mt‐rRNA) or transfer (mt‐tRNA) RNAs and are subsequently matured. Many of these events are described as occurring in discrete RNA granules close to the mtDNA in the mitochondrial matrix [3]. The early stages of mitochondrial ribosome assembly also occur within or close to these granules [4]. Apart from synthesis and maturation, much of the life cycle of mt‐mRNA is unclear [5]. Early *in vitro* studies support the notion that mt‐mRNA is first loaded onto the mammalian mitoribosomal 28S small subunit (mt‐SSU) [6, 7] but how is the nascent mt‐mRNA transported to the mt‐SSU and how is it protected ? Is mt‐mRNA protected by the mt‐SSU alone, or is stability dependent on further association with the mitoribosomal 39S large subunit (mt‐LSU) and associated factors? For example, the RNA‐binding complex formed by LRPPRC/SLIRP has been implicated in stabilizing mt‐mRNA that is not bound to the mitoribosome [8] and exposing its required sites for translation [9]. A recent report suggests SLIRP promotes correct association of mt‐mRNA with the mitoribosome [10]. Degradation of the antisense or mirror mt‐mRNAs clearly involves the mitochondrial PNPase in tandem with the hSUV3 helicase [11], but are these proteins also involved in sense mt‐mRNA degradation, and what promotes this degradation event ?

In an attempt to address some of these questions, we set out to selectively remove the mt‐LSU. To facilitate this, we have exploited the sequence specificity of mycobacterial endoribonuclease, VapC20 [12]. This PIN domain protein is part of the extensive toxin:antitoxin system that is found within *Mycobacterium tuberculosis*. It associates with the bacterial large ribosomal subunit and cleaves the 23S ribosomal RNA within the highly conserved sarcin:ricin loop (SRL), unless bound to its cognate VapB20 antitoxin counterpart [12, 13]. The exquisite cleavage selectivity is believed to be due to the ribonuclease recognizing the environment of SRL within the context of the ribosome, as VapC20 will not cleave isolated ribosomal RNA, *in vitro* or *in vivo* [12]. The SRL is essential during the elongation step of protein synthesis allowing the formation of stabilizing contacts between the ternary complex EF‐Tu/aa‐tRNA/GTP and the ribosome [14]. Being close to their GTPase domains, this rRNA loop interacts with both EF‐Tu and EF‐G [15, 16] and its cleavage differentially affects elongation factors binding to the bacterial ribosome [17]. The interaction of LSU rRNA with GTPases has also been described recently in human mitochondria, where the 16S mt‐rRNA domain VI, including the SRL and helices H96‐H97, interacts with human MTG1, a conserved GTPase that couples mt‐LSU assembly with intersubunit bridge formation [18].

Analysis revealed a strong but incomplete sequence identity between the SRL of *Escherichia coli* 23S rRNA and the human mitochondrial 16S rRNA. By fusing the VapC20 protein downstream of a mitochondrial localization signal, we have been able to show that, on induction, mtVapC20 localizes specifically to the mitochondrial matrix where it cleaves uniquely the mitoribosomal 16S rRNA not in the SRL, but in helix 91 that is immediately adjacent to the SRL, in the folded 16S mt‐rRNA. Expression of mtVapC20 caused the temporal and substantial depletion of the mt‐LSU without a concomitant decrease of mt‐SSU, resulting in the loss of almost all mt‐mRNA species tested. In many other systems that have been manipulated, levels of the LRPPRC/SLIRP complex reflect the steady‐state levels of most mt‐mRNA species, consistent with a mutual interdependency for stability [10, 19, 20, 21]. Under conditions of mtVapC20 expression, however, even with vanishingly low levels of mt‐mRNA, the LRPPRC/SLIRP complex is maintained. Notably, when mt‐SSU is depleted in tandem with mt‐LSU, mt‐mRNA levels as measured by the levels of *MTCO1* are partially recovered.

We hypothesize that monosome formation is required to promote the natural stability of the mt‐mRNA following its transfer from LRPPRC/SLIRP complex.

## Results

### mtVapC20 localizes to the human mitochondrial matrix

**Fig. 1:**
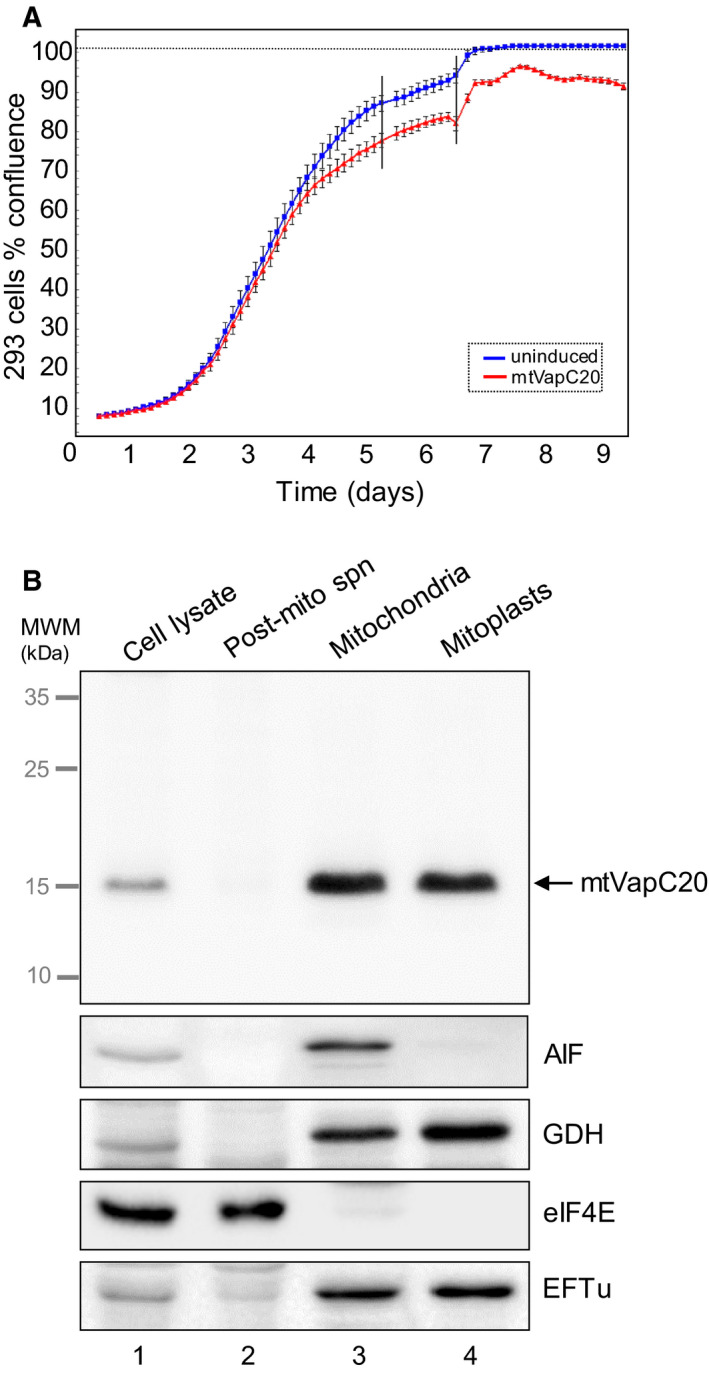
mtVapC20 expression and submitochondrial localization. (A) Growth was monitored for 9 days of both uninduced and induced mtVapC20 293 cells (*n* = 3) using the IncuCyte ZOOM^®^ System. Error bars represent the standard deviation of independent measures (*n* = 16) carried out every 3 h on each well. Black vertical bars denote the replacement of cell media to prevent acidification. (B) Submitochondrial localization of mtVapC20 was determined by immunoblotting of fractions as described. Anti‐6XHis antibody was used to detect mtVapC20 and the following markers for each mitochondrial compartment: apoptosis‐inducing factor (AIF: mitochondrial intermembrane space), glutamate dehydrogenase and mitochondrial elongation factor Tu (GDH and EFTu: mitochondrial matrix), and eukaryotic initiation factor 4E (eIF4E: cytosol). Data are representative of one experiment. MWM, molecular weight markers.

To determine whether VapC20 could cleave mitochondrial 16S rRNA, a Flp‐In™ 293 T‐REx cell line was engineered to inducibly express *M. tuberculosis* VapC20 fused in‐frame with an N‐terminal mitochondrial presequence from the *Neurospora crassa* ATP synthase subunit 9 (Su9; [22]) as described. Clones were isolated, and inducible mtVapC20 expression was confirmed. After 7 days of induction, the cell media acidified, consistent with an OXPHOS deficiency; however, following 9 days of induction cell growth was only mildly affected (Fig. 1A). To assess whether mitochondria‐targeted VapC20 (mtVapC20) was successfully imported into the mitochondrial matrix, mitochondria were isolated from an induced clonal line and subfractionated. The mtVapC20 displayed clear colocalization with markers of the mitochondrial matrix, glutamate dehydrogenase and mitochondrial elongation factor Tu (Fig. 1B). The product size (approx. 15 kDa, upper panel) was compatible with the cleavage of the Su9 presequence. Precursor was undetectable, consistent with a high level of import of the expressed product into mitochondria.

### mtVapC20 selectively cleaves the 16S mt‐rRNA

**Fig. 2:**
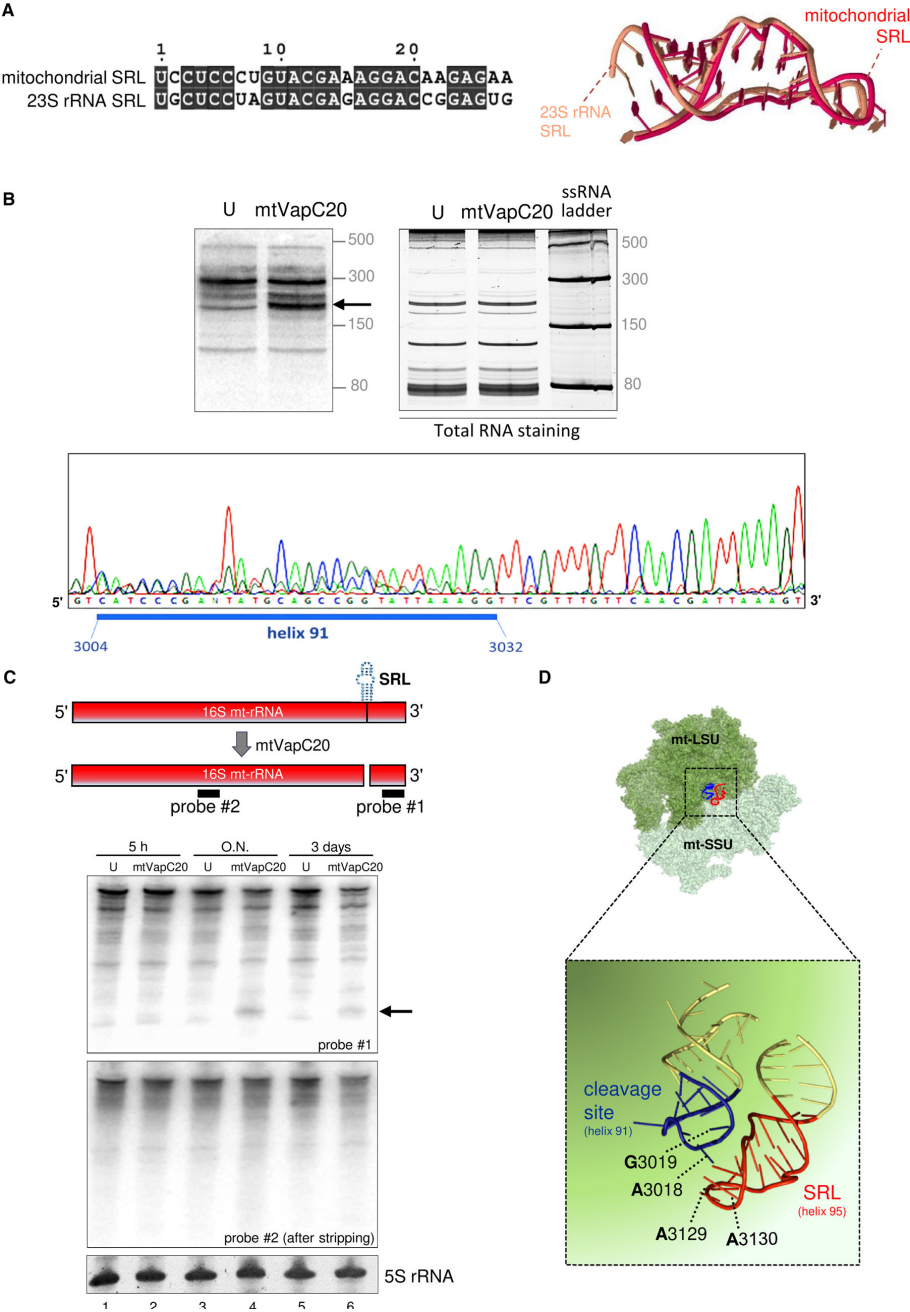
The sarcin:ricin loop and its cleavage by mtVapC20 in human mitochondrial 16S rRNA. (A) Sequences of the human mitochondrial 16S rRNA SRL (GenBank ID: NC_012920.1, 3115–3141) and bacterial 23S rRNA SRL (GenBank ID: NR_076322.1, 2648–2674) were aligned using the EMBL‐EBI MView online tool (https://www.ebi.ac.uk/Tools/msa/mview/). Identical nucleotides are blocked in black. Structural superimposition of mitochondrial (red) and bacterial (amber) SRL RNA tertiary structures was performed with the web server MultiSETTER [42] uploading PDB files (human 16S mt‐rRNA, PDB ID: 3J7Y; *E. coli* 23S SRL, PDB ID: 1Q9A) as input (*S*‐distance 0.209, *P*‐value 0.039). (B) Cleavage products generated following mtVapC20 expression were analysed by high‐resolution northern blot as described. Blots were hybridized with radiolabelled probes specific to 16S mt‐rRNA 3′ end. Upper left panel: mtVapC20 cleavage product is indicated with an arrow. Upper right panel: RNA was stained by SYBR™ Gold (Thermo Fisher Scientific) to validate loading. Low Range ssRNA Ladder (New England Biolabs) sizes are indicated on the right. Lower panel: sequence analysis of 5′‐end ligation‐mediated RT‐PCR product. Blue bar depicts the sequence of 16S mt‐rRNA helix 91. Data are representative of two independent experiments. (C) Cleavage analysis of 16S mt‐rRNA was performed with two different probes. Total RNA was extracted from uninduced (U) and mtVapC20 cells induced for 5 h, overnight (O.N.) and 3 days. The mtVapC20 cleavage product, only detected using probe #1, is indicated with an arrow. Data for each time point are representative of two independent experiments. 5S rRNA was used as loading control. (D) Magnification of the boxed area shows the mtVapC20 cleavage site and its proximity to the SRL. Structures of the assembled human mitoribosome (PDB ID: 3J9M; mt‐LSU in dark green, mt‐SSU in light green) and the cleavage site were obtained with pymol software (Open Source, Version 1.8.2.0, Schrödinger, New York, NY, USA).

Initial sequence and structure comparisons showed significant similarity between the *E. coli* 23S SRL and the mitochondrial 16S rRNA SRL (Fig. 2A); however, the region is not entirely conserved. To determine whether mtVapC20 was able to cleave the 16S mt‐rRNA, total cell RNA was isolated following a time course of induction. Northern blot analysis with probes covering 5′ and 3′ sequences revealed a unique product of approximately 200 nucleotides, detected specifically with the 3′ probe after 24 h of induction (Fig. 2B,C). Although specific cleavage occurred, the exact product size was unexpected, as cleavage in the SRL should generate a shorter 3′ product of 100 nucleotides. Trapping of the digestion product by 5′ blocked ligation‐mediated PCR and sequence analysis revealed a spectrum of products differing at their 5′ termini by a few bases but clearly indicating that cleavage occurred not at the SRL but at helix 91 (Fig. 2B, lower panel), shown to be only several angstroms away from the SRL after folding of the 16S mt‐rRNA (Fig. 2D, [23]). Interestingly, crystal structures of the bacterial ribosome show that helix 91 of the 23S rRNA establishes loop–loop tertiary interactions with SRL [24]. This tight conformation seems to be essential for both the assembly and the function of the large ribosomal subunit [25].

### Assembly of the mt‐SSU is independent of mt‐LSU formation

**Fig. 3:**
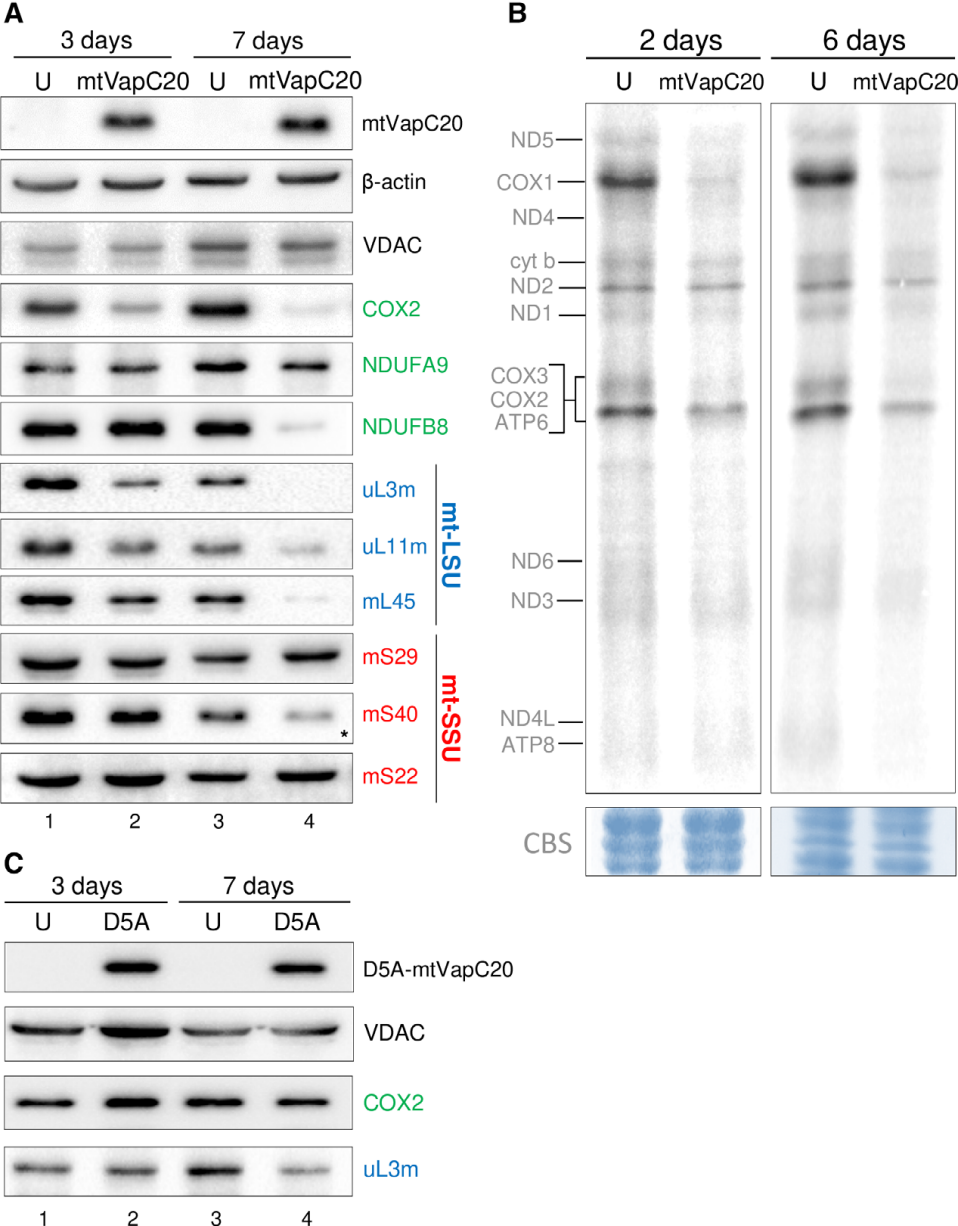
Effects of mtVapC20 expression on mitochondrial translation. (A) Cell lysates were prepared and aliquots (40 µg) subjected to immunoblotting as described. Steady‐state levels of representative OXPHOS subunits (green) were determined with antibodies against Complex I (NDUFA9 and NDUFB8) and Complex IV (COX2). Steady‐state levels of mt‐LSU (blue) and mt‐SSU (red) components were determined by western blot using antibodies against components of the mt‐LSU [HUGO protein names: MRPL3 (uL3m); MRPL11 (uL11m); MRPL45 (mL45)] and mt‐SSU [HUGO protein names: DAP3 (mS29); MRPS18B (mS40); MRPS22 (mS22)] as indicated. Asterisk indicates that the antibody possibly recognizes one of the two other mS40 isoforms, mL66 (MRPS18A), found in the mt‐LSU. Expression of mtVapC20 was detected by anti‐6XHis antibody. β‐actin and VDAC, a mitochondrial outer membrane protein, were used as loading control and mitochondrial marker, respectively. Data for each time point are representative of one experiment. (B) Metabolic ^35^S‐met/cys labelling of mitochondrial translation products was performed in uninduced (U) and mtVapC20 cells induced for 2 and 6 days as described. Mitochondrially encoded polypeptides were assigned after Chomyn [43]. Data for each time point are representative of one experiment. Equivalent protein loading was confirmed by Coomassie blue staining (CBS). (C) Immunoblotting analysis showing the effect of mutant mtVapC20 (D5A) expression on steady‐state levels of OXPHOS Complex IV, subunit 2 (COX2) and a constituent of the large mitoribosomal subunit (uL3m). Mutant mtVapC20 was detected with the anti‐6XHis antibody. VDAC was used as loading control. Data for each time point are representative of one experiment.

**Fig. 4:**
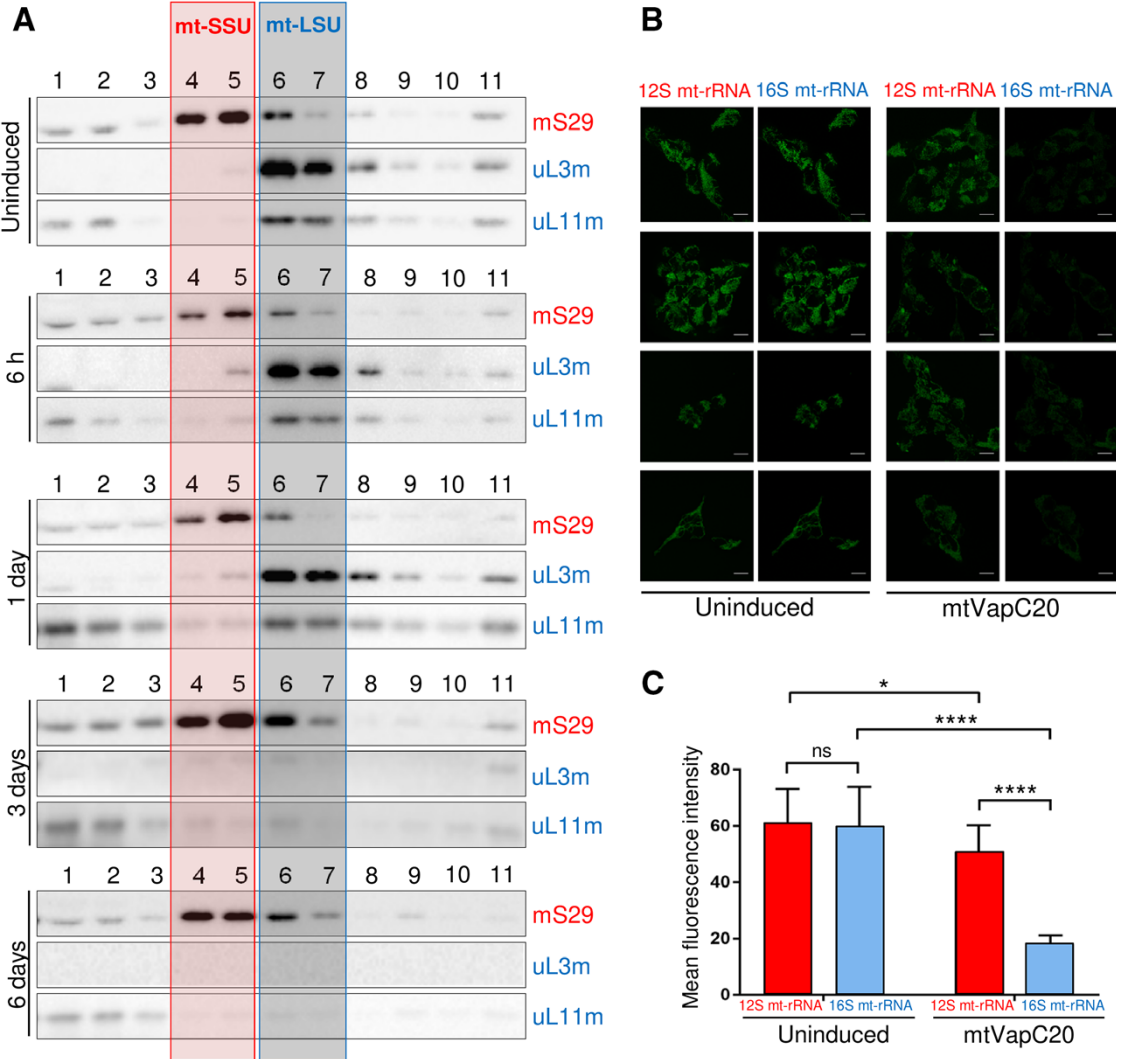
Effects of mtVapC20 expression on mitoribosomal components. (A) Isokinetic sucrose gradient centrifugation was used to analyse mitochondrial ribosomal protein distribution in mtVapC20 cells uninduced and induced for time periods as indicated. The migration of mt‐SSU (red box) and mt‐LSU (blue box) was determined using antibodies against mS29 (mt‐SSU), uL3m and uL11m (mt‐LSU). HUGO protein names: DAP3 (mS29); MRPL3 (uL3m); and MRPL11 (uL11m). Data are representative of two independent experiments. (B) Changes to distribution of 16S mt‐rRNA (in blue) and 12S mt‐rRNA (in red) in uninduced and mtVapC20‐expressing cells were determined by RNA FISH as described. For each sample, four representative confocal images are shown (scale bar: 10 µm). (C) Quantitative analysis of fluorescence intensities was performed using imagej and graphpad prism8 as detailed in Materials and methods. Results are presented as the mean ± SD (*n* = 9). Statistical analysis was performed using the Welch's *T* test (*P < 0.05; ****P < 0.0001).

To determine the effect of 16S mt‐rRNA cleavage, extracts were isolated at various stages postinduction and subjected to western or northern blot analysis. The mitochondrially encoded complex IV protein, COX2, was depleted by day 3 of induction and was markedly affected after 7 days (Fig. 3A). Similarly, NDUFB8, a nuclear‐encoded protein but a sensitive marker of complex I assembly, was also significantly depleted. Metabolic labelling of mitochondrial gene products was also substantially affected after 2 days of induction (Fig. 3B). These data are consistent with reduced mitochondrial protein synthesis. To determine the steady‐state levels of the mitochondrial ribosome, markers of the large (mt‐LSU) and small (mt‐SSU) subunit were assessed (Fig. 3A). Steady‐state levels of mt‐LSU components uL3m, uL11m and mL45 were depleted after 3 days but were markedly affected after 7 days, consistent with cleavage of the 16S mt‐rRNA leading to loss of mt‐LSU assembly. In contradistinction, mt‐SSU components mS29 and mS22 were unaffected, suggesting that mt‐SSU can assemble and remain stable in the absence of mt‐LSU. Targeting of the nuclease‐deficient D5A‐mtVapC20 mutant had no measurable effect on the levels of OXPHOS components or mitoribosome (Fig. 3C). To confirm that mt‐SSU assembly was unaffected, mtVapC20 was expressed for varying time periods and extracts were subjected to isokinetic sucrose gradient analysis. After 3 days of induction, fully assembled mt‐LSU was almost undetectable (Fig. 4A, fractions 6/7) with unassembled subunits being rapidly degraded or unexpressed. The mt‐SSU, however, is retained (compare signals for mS29 in fractions 4/5 of uninduced and 6 days mtVapC20 induction). To further confirm the specific loss of mt‐LSU on mtVapC20 induction, RNA FISH was performed with probes specific to either the 16S or 12S mt‐rRNA (Fig. 4B,C). After 6 days of induction, mtVapC20 led to a marked decrease in signal for 16S mt‐rRNA alone, consistent with the specific loss of mt‐LSU.

### Absence of mt‐LSU negatively affects mt‐mRNA levels

**Fig. 5:**
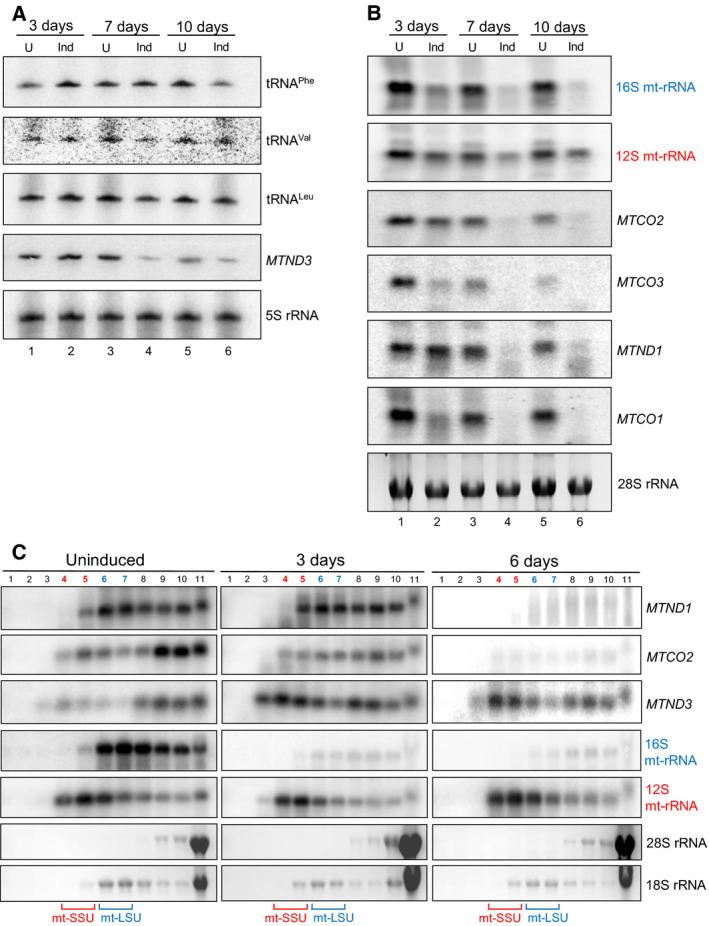
Effect of mtVapC20 expression on mitochondrially encoded RNAs. Steady‐state levels of mt‐RNAs were assessed by high‐resolution (A) and standard (B) northern blot as described. Total RNA was extracted from uninduced (U) and mtVapC20 cells induced (Ind) for 3, 7 and 10 days prior to analysis of RNA (2 µg per lane, A; 10 µg, B). Probes to cytosolic 5S rRNA and 28S rRNA were used as loading controls. (C) Isokinetic sucrose gradients (10–30%) were performed to analyse mitochondrial RNA distribution in uninduced and mtVapC20 cells induced for 3 and 6 days. RNA extracted from gradient fractions was analysed by northern blot with different probes, as indicated. Cytosolic 28S rRNA and 18S rRNA were used as loading controls. All the northern blot data relative to each different time point are representative of one experiment.

**Fig. 6:**
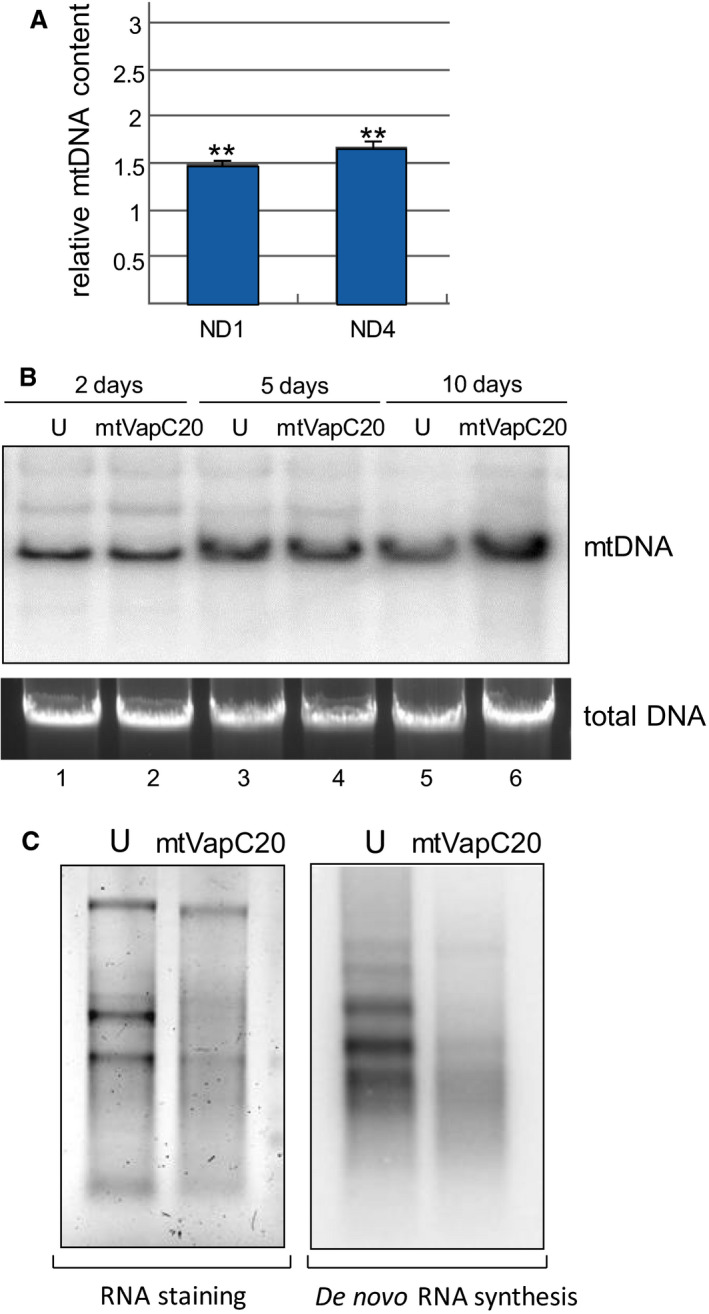
mtDNA levels and transcription in mtVapC20 cells. (A) Relative quantification of mitochondrial DNA levels by qPCR was performed after 10 days of induction. The relative mtDNA content of the induced mtVapC20 line was compared to uninduced control (set as equal to 1), normalized to 18S rRNA gene (endogenous control). Results are presented as the mean ± SD (*n* = 3). Statistical analysis was performed using one‐sample two‐tailed Student's *t* test (**P < 0.01). (B) Southern blot analysis was performed on total DNA isolated from uninduced (U) and cells induced (mtVapC20) for 2, 5 and 10 days. DNA (5 µg) was digested with BamHI, fractionated on a 0.7% agarose gel and electroblotted to nylon membrane. The filter was hybridized with radiolabelled probe for mitochondrial MTCO2 gene. Total DNA was stained by GelRed^®^ (Biotium, Fremont, CA, USA) as loading control. Data for each different time point are representative of one experiment. (C) *In organello* transcription assay was performed on uninduced (U) and mtVapC20 cells induced for 6 days. Total RNA was stained by SYBR™ Gold (Thermo Fisher Scientific) as loading control, and radiolabel detected as previously described. Data are representative of one experiment.

Previous data have shown that the depletion of mt‐SSU induced by a lack of modification or stability of the 12S mt‐rRNA in both mice models and in human cell lines does not promote increased degradation of most mt‐mRNAs [26, 27]. To assess whether mt‐mRNA is similarly protected when the mt‐LSU is absent, mtVapC20 was expressed for varying periods, after which RNA was isolated and northern blots performed (Fig. 5). Levels of analysed mt‐tRNAs were unaffected or only slightly reduced (Fig. 5A), consistent with the lack of any appreciable effect on mtDNA content (with exception for 10 days expression where mtDNA levels were increased) or transcription (Fig. 6). Further, 12S mt‐rRNA levels were only minimally depleted (Fig. 5B), reflecting the specificity of mtVapC20 cleavage and that mt‐SSU assembly was unaffected. Noticeably, however, although the pattern of steady‐state levels over the induction time course varied between mt‐RNA species, all mt‐mRNA species analysed showed decreases in steady‐state level by day 10. The assessed mt‐mRNA levels quantitatively reflected the decreasing levels shown for 16S mt‐rRNA, the marker of assembled mt‐LSU. *MTND3* was only mildly affected (Fig. 5A), even after 10 days of induction (approx. 40% of control). Thus, in the absence of mt‐LSU the majority of mt‐mRNA species appear to be selectively lost.

With the aim of analysing the association of mt‐mRNA with mitoribosomes, mtVapC20 was expressed for 3 or 6 days, extracts subjected to isokinetic sucrose gradient separation, followed by isolation of RNA from each fraction. Northern blot analysis (Fig. 5C) confirmed the presence of 12S mt‐rRNA that was normally associated with assembled mt‐SSU (fractions 4/5), even after 6 days of toxin expression. The 16S mt‐rRNA and mt‐mRNA species were strongly depleted and mainly associated with the remaining fraction of fully assembled monosome (6 days, fractions 8/9). The only exception was *MTND3* that showed a lower rate of decrease and a different distribution within the gradient after 3 days of induction, accumulating in the lighter fractions.

### LRPPRC/SLIRP is unaffected by the loss of mt‐LSU

**Fig. 7:**
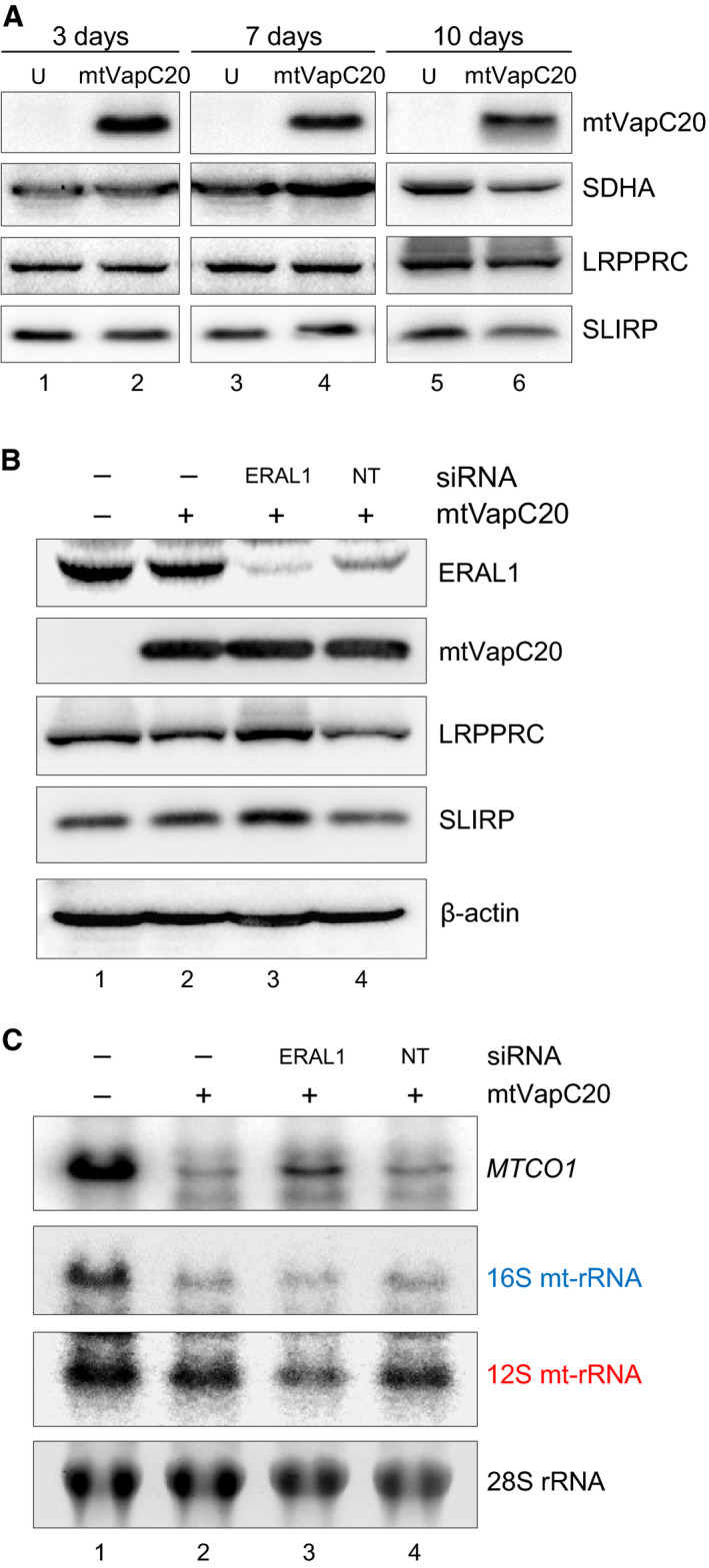
Effect of mtVapC20 expression on LRPPRC/SLIRP levels, and under conditions of mt‐SSU depletion. (A) mtVapC20 was induced for 3, 7 and 10 days. Cell lysates were prepared together with uninduced (U) controls. Levels of LRPPRC and SLIRP were determined by immunoblotting, and SDHA was used as loading control. Data for each different time point are representative of one experiment. (B, C) mtVapC20 was expressed for 4 days, concomitant with siRNA‐mediated depletion of ERAL1 or nontargeting (NT) control siRNA treatment. Cell lysates (B) and RNA (C) were prepared. Lysates were separated by 12% SDS/PAGE, and immunodetection revealed steady‐state levels of the proteins as indicated. β‐actin was used as loading control. RNA was subjected to northern blotting with probes to *MTCO1*, 16S mt‐rRNA and 12S mt‐rRNA to indicate changes in steady‐state levels. Probes to cytosolic 28S rRNA were used as loading control. Data obtained from both western and northern blots are representative of one experiment.

Evidence from patients, various transgenic mouse and human cultured cell models have implicated the LRPPRC/SLIRP RNA‐binding complex in the stability of mt‐mRNA [10, 19, 20, 21]. A substantial pool of data now shows a correlation between steady‐state levels of mt‐mRNA species and levels of LRPPRC. Increased steady‐state transcript levels have been shown to mirror increased LRPPRC levels as observed in mouse models depleted for the 12S mt‐rRNA methylase NSUN4 [28]. Several research groups have also reported that LRPPRC depletion led to decreased stability of most mt‐mRNA species in both cellular and animal models [8, 20, 29, 30]. Thus, upon mtVapC20 induction, LRPPRC levels would be predicted to decrease, to reflect the mitochondrial transcript reduction. Western blotting of extracts was performed following induction and showed that there was no measurable effect on the steady‐state levels of LRPPRC or SLIRP (Fig. 7A).

### Recovery of steady‐state levels of mt‐mRNA upon tandem depletion of mt‐LSU and mt‐SSU

One viable possibility to reconcile this large body of data with our observation is that in the absence of mt‐LSU, as facilitated by expression of mtVapC20, all mt‐SSU may be freely available and capable to receive mature mt‐mRNA from the LRPPRC/SLIRP complex but on transfer, there is no mt‐LSU to form the monosome, resulting in loss of protection for the mt‐mRNA leading to its rapid degradation. One prediction of this hypothesis is that concomitant loss of mt‐SSU and mt‐LSU should lead to restoration of transcript stability as the newly mature transcript would remain associated with the LRPPRC/SLIRP complex. To test this prediction, mt‐SSU levels were partially reduced by depleting the 12S mt‐rRNA chaperone, ERAL1. This was achieved by specific siRNA downregulation prior to the induction of mtVapC20. As shown in Fig. 7B, ERAL1 is depleted, resulting in less mt‐SSU as evidenced by the reduction in 12S mt‐rRNA (Fig. 7C, lane 3). Concomitantly, induction of mtVapC20 lowered the mt‐LSU levels, evidenced by 16S mt‐rRNA reduction (Fig. 7C, lanes 2–4). Although the reduction in mt‐SSU is only partial, there is a recovery in the steady‐state levels of *MTCO1* transcript (Fig. 7C, lane 3).

## Discussion

Targeting of the selective bacterial endoribonuclease VapC20 to human mitochondria results in the cleavage of 16S mt‐rRNA with the resultant loss of mt‐LSU. Unexpectedly, although the toxin has been shown previously to cleave *E. coli* 23S rRNA in exactly the same position in helix 95 as sarcin and other eukaryotic ribotoxins, mtVapC20 does not cleave the SRL of 16S mt‐rRNA in helix 95 but at helix 91, which is in close apposition to the SRL in the mitoribosome. *In vitro*, VapC20 is unable to cleave purified 23S rRNA alone, suggesting that cleavage requires a particular environment provided by the assembled or assembling ribosome. It would appear that in the case of the 16S mt‐rRNA, the SRL is recognized but actual cleavage occurs close to this site within the RNA. By targeting VapC20 to human mitochondria, we have shown that the mt‐SSU can assemble in the absence of the mt‐LSU. The work also suggests that the natural stability of mt‐mRNA shows dependence on the mt‐LSU, presumably due to it combining with the mt‐SSU to form the monosome, thus protecting the bound transcript.

Several previous reports have detailed the molecular consequences when mt‐LSU assembly has been partially compromised. Intriguingly, in certain cases where levels of mt‐mRNA were assessed, there was no evidence that mt‐mRNA stability was reduced. Two recent publications report the effect of depletion of the DDX28 helicase [4, 31], which partially affects mt‐LSU biogenesis. The maximal depletion of 16S mt‐rRNA, as a surrogate marker of assembled mt‐LSU, in each report did not fall below 40% levels of control. In contrast, longer term shRNA‐mediated depletion of the mt‐LSU component mL44 led to mitochondrial 16S rRNA levels dropping below 10% of control levels with a concomitant reduction of most mt‐mRNA [32], similar to our current report. One explanation for this apparent discrepancy is that depletion of DDX28 may cause aberrant mt‐LSU assembly, whereby monosome formation is still possible but translation is affected. This would also infer that levels of mt‐LSU were present in substantial excess over mt‐mRNA. Notably, our data showed that *MTND3* mRNA behaved in a different way. Even after prolonged mt‐LSU depletion (10 days of induction), this transcript was still present. In SLIRP KO mice, *MTND3* was the most affected transcript with respect to interactions with mitoribosomes [10]. Here, we report that in the absence of mt‐LSU, but in the presence of SLIRP, *MTND3* accumulated in the least dense fractions of the gradient, as did 12S mt‐rRNA (Fig. 5C). These observations may reflect the fact that *MTND3* is the shortest mt‐mRNA. It could be more easily protected by the mt‐SSU than longer transcripts by the LRPPRC/SLIRP complex as would be the case in mtVapC20 cells.

**Fig. 8:**
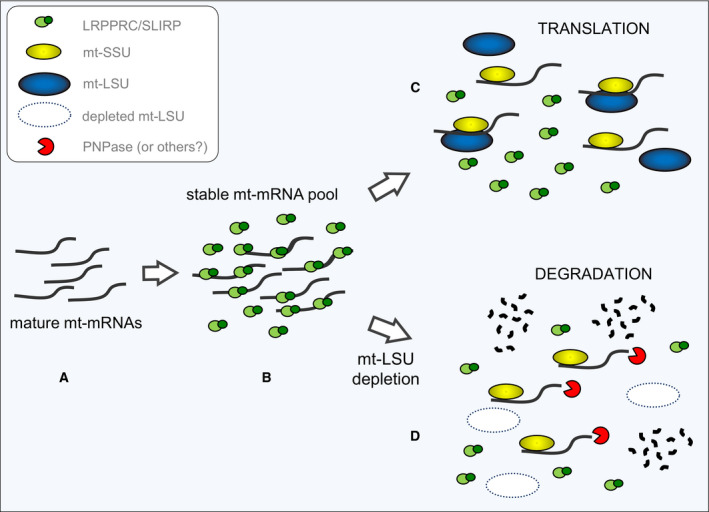
Model depicting mt‐mRNA fate in normal and mt‐LSU‐depleted cells. Cartoon showing processed and matured mt‐mRNAs within RNA granules (A). This model proposes that LRPPRC/SLIRP stabilizes mature mt‐mRNAs (B) and delivers them to either the mt‐SSU or the monosome (C). In this scenario, the mt‐mRNAs are translated. If mt‐LSU levels are low, as with mtVapC20 expression (D), mt‐mRNAs are still transferred to mt‐SSU but are degraded due to the lack of protection by the monosome.

There is mounting evidence that RNA maturation occurs within transient liquid droplets termed RNA granules and that this is likely to be where mitoribosomes are assembled [3, 4]. Many of the components required for processing and maturation of mammalian mt‐mRNA are now known, and many have been localized to these submitochondrial compartments [33]. However, although numerous mitoribosomal subunits have also been found close to the nucleoid, it is still unclear how the mature mt‐mRNA is loaded onto the mitoribosome or indeed where this occurs. Early *in vitro* studies supported the idea that mt‐mRNA is first loaded onto the mt‐SSU before monosome formation [6, 7]. It has been also speculated that the monosome can form without mt‐mRNA loaded onto it and that the mRNA is inserted later as with leaderless bacterial mRNAs [34, 35]. This hypothesis implies that LRPPRC/SLIRP may load the mt‐mRNA directly onto the monosome, not onto the mt‐SSU. We believe our data are now consistent with a model whereby the LRPPRC/SLIRP complex associates with newly processed mt‐mRNAs and ensures normal maturation through regulated polyadenylation also ensuring normal translation by transferring the mature mt‐mRNA to the mt‐SSU or to the monosome (Fig. 8).

It has previously been shown that POLRMT knockout causes decrease of *de novo* mitochondrial transcription with a concomitant reduction of LRPPRC levels [21]. Although our data do not completely rule out a negative effect of mt‐LSU lack on mitochondrial transcription, the unchanged levels of LRPPRC in VapC20 cells suggest that the selective loss of mt‐mRNAs depends on their degradation rather than their diminished synthesis as a result of mt‐LSU depletion. Hence, correct stability and translation are driven by monosome formation with the mt‐LSU (Fig. 8). In the absence of LRPPRC/SLIRP, mt‐mRNA can be degraded before associating with mitoribosomal subunits, with the remainder being aberrantly polyadenylated or aberrantly loaded onto the mitoribosome, resulting in translational abnormalities.

Finally, the inducible VapC20 cell line reported here could provide a tool for studying processes related to mitochondrial gene expression in humans. It may have potential to clarify some central aspects of mt‐mRNA turnover and the identification of RNases involved in quality control of untranslated transcripts.

## Materials and methods

### Tissue culture, constructs and transfections

293 Flp‐In T‐REx cells (Thermo Fisher Scientific, Carlsbad, CA, USA) were cultured (37 °C, humidified 5% CO_2_) in Dulbecco's modified Eagle's medium supplemented with 10% (v/v) fetal calf serum, 50 µg·mL^−1^ uridine, 1× nonessential amino acids and 10 µg·mL^−1^ Blasticidin^S^. Stable transfections were carried out using the constructs described below and Superfect (Qiagen, Hilden, Germany) following manufacturer's recommendations. Post‐transfection selection was performed with 100 µg·mL^−1^ Hygromycin^B^.

The construct to facilitate inducible expression of full‐length C‐terminal 6xHis‐tagged VapC20 (UniProtKB ID: P95004) was prepared by generating an amplicon using the plasmid pKW332549c [12] as a template and the following primers: For 5′‐CACACAGCGCGCCTACTCTTCCATGATCTTCGTCGACACGTC‐3′ and Rev 5′‐ATTGCGGCCGCCTAATGGTGATGGTGATGGT‐3′. The amplicon was BssHII/Not1 digested and ligated into pcDNA5/FRT/TO plasmid carrying the *N. crassa* ATP synthase subunit 9 presequence, generating a Su9‐VapC20‐His fusion protein (mtVapC20) that was internalized into the mitochondrial matrix.

The nontoxic D5A‐mtVapC20 construct was obtained by site‐directed mutagenesis, performed with the QuikChange II Site‐Directed Mutagenesis Kit (Stratagene, San Diego, CA, USA), following manufacturer's guidelines. This mutant construct was generated with the following primers: D5A‐sense 5′‐TCCATGATCTTCGTCGCGACGTCCTTCTGGGCT‐3′, D5A‐antisense 5′‐AGCCCAGAAGGACGTCGCGACGAAGATCATGGA‐3′, and pcDNA5/FRT/TO/Su9‐VapC20‐His as the template. Both the wild‐type and mutated constructs were validated by sequencing of both DNA strands.

ERAL1 siRNA transfections were performed as described in Ref. [26] on approximately 12 000 mtVapC20 inducible cells per cm^2^ with Lipofectamine RNAiMAX Transfection Reagent (Thermo Fisher Scientific) in Optimem‐I medium (33 nm siRNA). Custom and control nontargeting (NT) duplex siRNAs were purchased pre‐annealed from Eurogentec (Liege, Belgium): ERAL1sense 5′‐GUGUCCUGGUCAUGAACAAdTdT‐3′; ERAL1antisense 5′‐UUGUUCAUGACCAGGACACdTdT‐3′.

For the growth curve analyses, 293 cells that could inducibly express mtVapC20 were seeded onto 6‐well plates, induced with 1 µg·mL^−1^ tetracycline or uninduced as controls, and propagated with the IncuCyte ZOOM^®^ System (Essen BioScience, Ann Arbor, MI, USA). Sixteen fields per well, with biological triplicates, were imaged every 3 h for 9 days. All images were analysed by the incucyte zoom Software.

### Cellular and mitochondrial subfractionation

Subcellular fractions were prepared as described previously [36] with few modifications. Cells expressing mtVapC20 were harvested, resuspended in HB (0.6 m mannitol, 10 mm Tris/HCl, pH 7.4, 1 mm EGTA), supplemented with 0.1% BSA (w/v), and subjected to standard differential centrifugation. Mitochondria were finally pelleted (11 000 ***g***, 10 min, 4 °C) and resuspended in HB. The post‐mitochondrial supernatant (cytosolic fraction) was retained after centrifugation. To obtain submitochondrial fractions, mitochondria (300 µg) were treated with proteinase K (PK, 1.5 µg) on ice for 30 min followed by addition of 5 mm PMSF, pelleted (11 000 ***g***, 10 min, 4 °C) and resuspended in HB. Mitoplasts were obtained resuspending PK‐treated mitochondria in 9 volumes of 10 mm Tris/HCl, pH 7.4, and treated with PK as described above. Proteins (40 µg) from whole cell lysate, cytosolic and mitochondrial fractions were separated by 12% SDS/PAGE, transferred to PVDF membrane and analysed by immunoblotting.

### Northern blotting analyses

Northern blots were performed as described previously [37]. Briefly, cells expressing mtVapC20 were harvested at different time points (3, 7 and 10 days) and total RNA was extracted. Aliquots (10 µg) were electrophoresed through 1.2% agarose under denaturing conditions and transferred to GeneScreen Plus membrane (Perkin Elmer, Waltham, MA, USA) following the manufacturer's protocol. Radiolabelled probes were generated using random hexamers on PCR‐generated templates corresponding to internal regions of the relevant genes.

High‐resolution northerns were carried out to analyse the 16S mt‐rRNA cleavage product and mt‐tRNAs. Total RNA (2 µg) was denatured in 40% formamide/5 mm EDTA at 95 °C, 5 min before being separated through 15% urea polyacrylamide gels in 1× TBE (100 mm Tris base, 100 mm boric acid, 2 mm EDTA disodium salt). RNA was transferred to GeneScreen Plus membrane in 0.25× TBE at 150 mA, 15 min followed by 500 mA for 30 min after which the membrane was exposed to UV (optimal crosslink, Stratalinker, Stratagene). Hybridization conditions and probes were as described in [38]. Signals were detected by Typhoon FLA 9500 phosphorimager (GE Healthcare Life Sciences, Marlborough, MA, USA) and quantified by imagequant tl 8.1 software (GE Healthcare Life Sciences). To detect the RNA fragment produced by mtVapC20 cleavage, the following primers were used that were specific to the 3′‐end of 16S mt‐rRNA: SRLdown‐For 5′‐TAAGGCCTACTTCACAAAGCG‐3′; SRLdown‐Rev 5′‐CTTGGGTGGGTGTGGGTATAA‐3′.

The analysis to discriminate the mitochondrial 16S rRNA cleavage product was carried out by high‐resolution northern blot as described above with few modifications. Briefly, uninduced and mtVapC20‐expressing cells were harvested at different time points, and total RNA was extracted and 2 µg electrophoresed through a 4.5% urea polyacrylamide gel in 1× TBE. For the detection of 16S mt‐rRNA, two different probes were used, generated with the following primer pairs: SRLdown‐For and SRLdown‐Rev (probe #1); 16S‐For 5′‐CCAATTAAGAAAGCGTTCAAG‐3′ and 16S‐Rev 5′‐CATGCCTGTGTTGGGTTGACA‐3′ (probe #2, upstream of expected cleavage site).

### 5′‐end ligation‐mediated RT‐PCR

Total RNA (10 µg) was phosphorylated by T4 PNK (New England Biolabs, Ipswich, MA, USA) in the presence of 1 mm ATP to ensure ligatable ends post‐mtVapC20 cleavage. Phosphorylated RNAs of sizes between 150 and 300 nucleotides were fractionated and gel‐purified on a 10% PAA/8 m urea gel. Eluted RNA fragments were ligated with 20 pmol of RNA linker (L5, 5′OH‐AGGGAGGACGAUGCGG‐3′OH) in the recommended buffer by 20 Units T4 RNA Ligase (New England Biolabs) at 37 °C for 3 h. The reaction was subjected to phenol/chloroform extraction and sodium acetate/ethanol precipitation. The product was reverse transcribed using the Superscript II enzyme (Thermo Fisher Scientific) primed with 10 pmol of SRLdown‐Rev oligonucleotide.

An aliquot (10%) of this cDNA was used to programme a standard PCR reaction [0.5 mm dNTPs, 2 mm MgCl_2_, 3 Units Taq Polymerase (Thermo Fisher Scientific)], with 10 pmol of P5‐For (5′‐AGGGAGGACGATGCGG‐3′) and 10 pmol of SRLdown‐Rev. A PCR single product (~ 200 nt) was purified using E.Z.N.A. gel extraction kit (Omega Bio‐Tek, Norcross, GA, USA) prior to DNA sequencing using BigDye terminator cycle sequencing chemistry (Applied Biosystems, Foster City, CA, USA) on the ABI PRISM 3130xl Genetic Analyzer (Thermo Fisher Scientific). Cycle sequencing reactions were carried out using 20 ng of purified fragment as template primed with 10 pmol of the internal oligonucleotide INT‐SRLdown‐Rev (5′‐GAAGGCGCTTTGTGAAGTAGG‐3′).

### Immunoblotting

Western blots were performed and developed as described in Ref. [39] using antibodies as follows: 6XHis, VDAC, uL3m, mS29, SLIRP, COX2, NDUFA9, NDUFB8 (Abcam, Cambridge, UK); AIF, uL11m (New England Biolabs); eIF4E (Cell Signaling Technology, Danvers, MA, USA); SDHA (Thermo Fisher Scientific); mL45, mS40, mS22 (ProteinTech Group, Rosemont, IL, USA); LRPPRC (Santa Cruz Biotechnology, Dallas, TX, USA); β‐actin (Sigma‐Aldrich, St. Louis, MO, USA); and GDH, EFTu (custom made).

### *De novo* mitochondrial protein synthesis

Metabolic labelling of *de novo* mitochondrial protein synthesis in cultured cells was performed after 2 and 6 days of mtVapC20 induction and carried out as described by [40] incubating cells with [^35^S] methionine/cysteine (Perkin Elmer) for 1 h. Aliquots (50 µg) of total cell protein were separated by 15% SDS/PAGE and radioactive signals were detected as described above.

### Isokinetic sucrose gradient analysis

Cell lysates [1 mg in lysis buffer (50 mm Tris/HCl, pH 7.4, 150 mm NaCl, 1 mm EDTA, 1% Triton X‐100, protease inhibitor cocktail, 1 mm PMSF and 10 mm MgCl_2_)] were prepared at different time points of mtVapC20 induction and loaded onto a linear sucrose gradient [1 mL, 10–30% (v/v)] in 50 mm Tris/HCl, pH 7.2, 10 mm MgOAc, 40 mm NH_4_Cl, 100 mm KCl, 1 mm PMSF and 50 μg·mL^−1^ chloramphenicol. Gradients were subjected to centrifugation in a OptimaTLX bench ultracentrifuge (Beckman Coulter, Brea, CA, USA), using a TLS55 rotor at 39 000 r.p.m. for 135 mins at 4 °C. Eleven fractions (100 μL) were collected and analysed by immunoblot.

### Southern blotting and qPCR analysis

Southern blotting was performed as previously described [36]. The *MTCO2* probe was generated by PCR using the following primers: COX2‐For 5′‐CCTAGAACCAGGCGAC‐3′ and COX2‐Rev 5′‐GTCGTGTAGCGGTGAA‐3′, spanning residues 7966–8150 of the mtDNA reference sequence (GenBank ID: NC_012920.1). Radioactive signals were detected using a Typhoon FLA 9500 PhosphorImager (GE Healthcare Life Sciences).

To detect levels of mtDNA by quantitative PCR, the following primer pairs were used to amplify a sequence within each target gene: ND1‐For 5′‐AATCGCAATGGCATTCC‐3′ and ND1‐Rev 5′‐CGATGGTGAGAGCTAAGG‐3′, ND4‐For 5′‐CCATTCTCCTCCTATCCCTCAAC‐3′ and ND4‐Rev 5′‐CACAATCTGATGTTTTGGTTAAACTATATTT‐3′. Cytosolic 18S rRNA was used as a reference: 18S‐For 5′‐GTAACCCGTTGAACCCCATT‐3′ and 18S‐Rev 5′‐CCATCCAATCGGTAGTAGCG‐3′.

### *In organello* RNA synthesis

The transcription assay was performed on mitochondria isolated from uninduced and mtVapC20‐expressing cells (6 days of induction) as previously described [41] with few modifications. Briefly, mitochondria (320 µg) were incubated with 10 µCi of [α‐^32^P]UTP (800 Ci·mmol^−1^; Perkin Elmer) for 1 h at 37 °C with gentle rotation. Mitochondria were then washed twice in washing buffer (10 mm Tris/HCl, pH 6.8, 0.15 mm MgCl_2_ and 10% glycerol) and pelleted by centrifugation at 12 000 ***g*** for 3 min at 4 °C. Pellets were resuspended in 1 mL of TRIzol^®^ Reagent (Thermo Fisher Scientific) and RNA was extracted.

To analyse the products of the *in organello* transcription, RNA was fractionated on a 2.2 m formaldehyde/1.4% agarose gel and stained by SYBR™ Gold (Thermo Fisher Scientific) to validate loading. The gel was dried and exposed to a PhosphorImager screen, and radioactive signals were detected as described above.

### Mitochondrial rRNA FISH

Cells capable of inducibly expressing mtVapC20 were seeded onto coverslips in a 6‐well plate, induced with 1 µg·mL^−1^ tetracycline or left uninduced as controls. At 20–30% confluency, cells were fixed using 4% PFA/0.2% glutaraldehyde (10 min, room temperature) and washed twice in 1× PBS. Cells were then permeabilized (70% ethanol, 1 h, room temperature), washed twice with 1× PBS and equilibrated in 1 mL of wash buffer (10% formamide, 2× SSC) for 5 min. 16S‐ and 12S mt‐rRNA Stellaris^®^ Probe Sets (Biosearch Technologies, Novato, CA, USA) were diluted in TE Buffer (10 mm Tris/HCl, 1 mm EDTA pH 7.5) to 12.5 µm, then added 1 : 100 to hybridization buffer (10% formamide, 10% dextran sulfate, 2× SSC). Droplets (100 µL) of probe‐hybridization buffer solution were placed onto parafilm in a humidified chamber on to which coverslips were lowered and incubated overnight at 37 °C in a humidified atmosphere. Coverslips were rinsed, incubated twice in wash buffer (30 min), then stored in 1× PBS until mounted in ProLong^®^ Diamond Antifade Mountant. Confocal imaging was performed using a SP8 microscope (Leica, Wetzlar, Germany) fitted with a Leica HyD hybrid detector and imagej software (NIH, Bethesda, MD, USA) was used to quantify fluorescence intensity. Each channel was analysed independently from maximum intensity projections generated from acquired stacks. To reduce background interference during intensity calculations, an area was segmented around the high‐intensity signal using thresholding. In all cases, the area was determined by thresholding the 12S mt‐rRNA image. imagej was then used to measure the mean intensity for both channels using this area. The measurements were analysed using graphpad prism8 software (San Diego, CA, USA).

## Conflict of interest

The authors declare no conflict of interest.

## Author contributions

FB performed all the experiments (with exception of RNA FISH). YP‐K carried out RNA FISH and associated data analysis. ZMC‐L and RNL designed the study and provided funding. ZMC‐L, RNL and FB supervised the experimental design and data analysis and wrote the manuscript.

## References

[1] Montoya J , Christianson T , Levens D , Rabinowitz M & Attardi G (1982) Identification of initiation sites for heavy‐strand and light‐strand transcription in human mitochondrial DNA. Proc Natl Acad Sci USA 79, 7195–7199. 6185947 10.1073/pnas.79.23.7195 PMC347305

[2] Gustafsson CM , Falkenberg M & Larsson N‐G (2016) Maintenance and expression of mammalian mitochondrial DNA. Annu Rev Biochem 85, 133–160. 27023847 10.1146/annurev-biochem-060815-014402

[3] Jourdain AA , Boehm E , Maundrell K & Martinou JC (2016) Mitochondrial RNA granules: compartmentalizing mitochondrial gene expression. J Cell Biol 212, 611–614. 26953349 10.1083/jcb.201507125 PMC4792075

[4] Antonicka H & Shoubridge EA (2015) Mitochondrial RNA granules are centers for posttranscriptional RNA processing and ribosome biogenesis. Cell Rep 10, 920–932. 25683715 10.1016/j.celrep.2015.01.030

[5] Rackham O , Busch JD , Matic S , Siira SJ , Kuznetsova I , Atanassov I , Ermer JA , Shearwood AMJ , Richman TR , Stewart JB *et al* (2016) Hierarchical RNA processing is required for mitochondrial ribosome assembly. Cell Rep 16, 1874–1890. 27498866 10.1016/j.celrep.2016.07.031

[6] Liao HX & Spremulli LL (1989) Interaction of bovine mitochondrial ribosomes with messenger RNA. J Biol Chem 264, 7518–7522. 2540195

[7] Christian BE & Spremulli LL (2010) Preferential selection of the 5′‐terminal start codon on leaderless mRNAs by mammalian mitochondrial ribosomes. J Biol Chem 285, 28379–28386. 20610392 10.1074/jbc.M110.149054 PMC2934702

[8] Sasarman F , Brunel‐Guitton C , Antonicka H , Wai T & Shoubridge EA (2010) LRPPRC and SLIRP interact in a ribonucleoprotein complex that regulates posttranscriptional gene expression in mitochondria. Mol Biol Cell 21, 1315–1323. 20200222 10.1091/mbc.E10-01-0047 PMC2854090

[9] Siira SJ , Spåhr H , Shearwood AMJ , Ruzzenente B , Larsson NG , Rackham O & Filipovska A (2017) LRPPRC‐mediated folding of the mitochondrial transcriptome. Nat Commun 8, 1532. 29146908 10.1038/s41467-017-01221-z PMC5691074

[10] Lagouge M , Mourier A , Lee HJ , Spåhr H , Wai T , Kukat C , Silva Ramos E , Motori E , Busch JD , Siira S *et al* (2015) SLIRP regulates the rate of mitochondrial protein synthesis and protects LRPPRC from degradation. PLoS Genet 11, e1005423. 26247782 10.1371/journal.pgen.1005423 PMC4527767

[11] Borowski LS , Dziembowski A , Hejnowicz MS , Stepien PP & Szczesny RJ (2013) Human mitochondrial RNA decay mediated by PNPase‐hSuv3 complex takes place in distinct foci. Nucleic Acids Res 41, 1223–1240. 23221631 10.1093/nar/gks1130 PMC3553951

[12] Winther KS , Brodersen DE , Brown AK & Gerdes K (2013) VapC20 of *Mycobacterium tuberculosis* cleaves the sarcin‐ricin loop of 23S rRNA. Nat Commun 4, 2796. 24225902 10.1038/ncomms3796

[13] Deep A , Kaundal S , Agarwal S , Singh R & Thakur KG (2017) Crystal structure of *Mycobacterium tuberculosis* VapC20 toxin and its interactions with cognate antitoxin, VapB20, suggest a model for toxin–antitoxin assembly. FEBS J 284, 4066–4082. 28986943 10.1111/febs.14289

[14] Lee TH , Blanchard SC , Kim HD , Puglisi JD & Chu S (2007) The role of fluctuations in tRNA selection by the ribosome. Proc Natl Acad Sci USA 104, 13661–13665. 17699629 10.1073/pnas.0705988104 PMC1949337

[15] Martin Schmeing T , Voorhees RM , Kelley AC , Gao YG , Murphy FV IV , Weir JR & Ramakrishnan V (2009) The crystal structure of the ribosome bound to EF‐Tu and aminoacyl‐tRNA. Science 326, 688–694. 19833920 10.1126/science.1179700 PMC3763470

[16] Gao YG , Selmer M , Dunham CM , Weixlbaumer A , Kelley AC & Ramakrishnan V (2009) The structure of the ribosome with elongation factor g trapped in the posttranslocational state. Science 326, 694–699. 19833919 10.1126/science.1179709 PMC3763468

[17] García‐Ortega L , Álvarez‐García E , Gavilanes JG , Martínez‐del‐Pozo Á & Joseph S (2010) Cleavage of the sarcin‐ricin loop of 23S rRNA differentially affects EF‐G and EF‐Tu binding. Nucleic Acids Res 38, 4108–4119. 20215430 10.1093/nar/gkq151 PMC2896532

[18] Kim HJ & Barrientos A (2018) MTG1 couples mitoribosome large subunit assembly with intersubunit bridge formation. Nucleic Acids Res 46, 8435–8453. 30085276 10.1093/nar/gky672 PMC6144824

[19] Sasarman F , Nishimura T , Antonicka H , Weraarpachai W , Shoubridge EA , Allen B , Burelle Y , Charron G , Coderre L , DesRosiers C *et al* (2015) Tissue‐specific responses to the LRPPRC founder mutation in French Canadian Leigh Syndrome. Hum Mol Genet 24, 480–491. 25214534 10.1093/hmg/ddu468 PMC4275074

[20] Ruzzenente B , Metodiev MD , Wredenberg A , Bratic A , Park CB , Cámara Y , Milenkovic D , Zickermann V , Wibom R , Hultenby K *et al* (2012) LRPPRC is necessary for polyadenylation and coordination of translation of mitochondrial mRNAs. EMBO J 31, 443–456. 22045337 10.1038/emboj.2011.392 PMC3261557

[21] Kühl I , Miranda M , Posse V , Milenkovic D , Mourier A , Siira SJ , Bonekamp NA , Neumann U , Filipovska A , Polosa PL *et al* (2016) POLRMT regulates the switch between replication primer formation and gene expression of mammalian mtDNA. Sci Adv 2, e1600963. 27532055 10.1126/sciadv.1600963 PMC4975551

[22] Viebrock A , Perz A & Sebald W (1982) The imported preprotein of the proteolipid subunit of the mitochondrial ATP synthase from *Neurospora crassa*. Molecular cloning and sequencing of the mRNA. EMBO J 1, 565–571. 6329691 10.1002/j.1460-2075.1982.tb01209.x PMC553088

[23] Brown A , Amunts A , Bai XC , Sugimoto Y , Edwards PC , Murshudov G , Scheres SHW & Ramakrishnan V (2014) Structure of the large ribosomal subunit from human mitochondria. Science 346, 718–722. 25278503 10.1126/science.1258026 PMC4246062

[24] Ban N , Nissen P , Hansen J , Moore PB & Steitz TA (2000) The complete atomic structure of the large ribosomal subunit at 2.4 Å resolution. Science 289, 905–920. 10937989 10.1126/science.289.5481.905

[25] Lancaster L , Lambert NJ , Maklan EJ , Horan LH & Noller HF (2008) The sarcin‐ricin loop of 23S rRNA is essential for assembly of the functional core of the 50S ribosomal subunit. RNA 14, 1999–2012. 18755834 10.1261/rna.1202108 PMC2553751

[26] Dennerlein S , Rozanska A , Wydro M , Chrzanowska‐Lightowlers ZMA & Lightowlers RN (2010) Human ERAL1 is a mitochondrial RNA chaperone involved in the assembly of the 28S small mitochondrial ribosomal subunit. Biochem J 430, 551–558. 20604745 10.1042/BJ20100757 PMC2995420

[27] Metodiev MD , Lesko N , Park CB , Cámara Y , Shi Y , Wibom R , Hultenby K , Gustafsson CM & Larsson NG (2009) Methylation of 12S rRNA is necessary for in vivo stability of the small subunit of the mammalian mitochondrial ribosome. Cell Metab 9, 386–397. 19356719 10.1016/j.cmet.2009.03.001

[28] Metodiev MD , Spåhr H , Loguercio Polosa P , Meharg C , Becker C , Altmueller J , Habermann B , Larsson NG & Ruzzenente B (2014) NSUN4 is a dual function mitochondrial protein required for both methylation of 12S rRNA and coordination of mitoribosomal assembly. PLoS Genet 10, e1004110. 24516400 10.1371/journal.pgen.1004110 PMC3916286

[29] Cooper MP , Qu L , Rohas LM , Lin J , Yang W , Erdjument‐Bromage H , Tempst P & Spiegelman BM (2006) Defects in energy homeostasis in Leigh syndrome French Canadian variant through PGC‐1α/LRP130 complex. Genes Dev 20, 2996–3009. 17050673 10.1101/gad.1483906 PMC1620022

[30] Gohil VM , Nilsson R , Belcher‐Timme CA , Luo B , Root DE & Mootha VK (2010) Mitochondrial and nuclear genomic responses to loss of LRPPRC expression. J Biol Chem 285, 13742–13747. 20220140 10.1074/jbc.M109.098400 PMC2859537

[31] Tu YT & Barrientos A (2015) The human mitochondrial DEAD‐box protein DDX28 resides in RNA granules and functions in mitoribosome assembly. Cell Rep 10, 854–864. 25683708 10.1016/j.celrep.2015.01.033 PMC4534351

[32] Yeo JHC , Skinner JPJ , Bird MJ , Formosa LE , Zhang JG , Kluck RM , Belz GT & Chong MMW (2015) A role for the mitochondrial protein Mrpl44 in maintaining OXPHOS capacity. PLoS ONE 10, e0134326. 26221731 10.1371/journal.pone.0134326 PMC4519308

[33] Pearce SF , Rebelo‐Guiomar P , D'Souza AR , Powell CA , Van Haute L & Minczuk M (2017) Regulation of mammalian mitochondrial gene expression: recent advances. Trends Biochem Sci 42, 625–639. 28285835 10.1016/j.tibs.2017.02.003 PMC5538620

[34] Lightowlers RN , Rozanska A & Chrzanowska‐Lightowlers ZM (2014) Mitochondrial protein synthesis: figuring the fundamentals, complexities and complications, of mammalian mitochondrial translation. FEBS Lett 588, 2496–2503. 24911204 10.1016/j.febslet.2014.05.054 PMC4099522

[35] Gopalakrishna S , Pearce SF , Dinan AM , Schober FA , Cipullo M , Spåhr H , Khawaja A , Maffezzini C , Freyer C , Wredenberg A *et al* (2019) C6orf203 is an RNA‐binding protein involved in mitochondrial protein synthesis. Nucleic Acids Res 47, 9386–9399. 31396629 10.1093/nar/gkz684 PMC6755124

[36] Bruni F , Gramegna P , Oliveira JMA , Lightowlers RN & Chrzanowska‐Lightowlers ZMA (2013) REXO2 is an oligoribonuclease active in human mitochondria. PLoS ONE 8, e64670. 23741365 10.1371/journal.pone.0064670 PMC3669425

[37] Chrzanowska‐Lightowlers ZMA , Preiss T & Lightowlers RN (1994) Inhibition of mitochondrial protein synthesis promotes increased stability of nuclear‐encoded respiratory gene transcripts. J Biol Chem 269, 27322–27328. 7525553

[38] Rorbach J , Yusoff AA , Tuppen H , Abg‐Kamaludin DP , Chrzanowska‐Lightowlers ZMA , Taylor RW , Turnbull DM , Mcfarland R & Lightowlers RN (2008) Overexpression of human mitochondrial valyl tRNA synthetase can partially restore levels of cognate mt‐tRNAVal carrying the pathogenic C25U mutation. Nucleic Acids Res 36, 3065–3074. 18400783 10.1093/nar/gkn147 PMC2396425

[39] Soleimanpour‐Lichaei HR , Kühl I , Gaisne M , Passos JF , Wydro M , Rorbach J , Temperley R , Bonnefoy N , Tate W , Lightowlers R *et al* (2007) mtRF1a is a human mitochondrial translation release factor decoding the major termination codons UAA and UAG. Mol Cell 27, 745–757. 17803939 10.1016/j.molcel.2007.06.031 PMC1976341

[40] Wydro M , Bobrowicz A , Temperley RJ , Lightowlers RN & Chrzanowska‐Lightowlers ZM (2010) Targeting of the cytosolic poly(A) binding protein PABPC1 to mitochondria causes mitochondrial translation inhibition. Nucleic Acids Res 38, 3732–3742. 20144953 10.1093/nar/gkq068 PMC2887948

[41] Enríquez JA , Pérez‐Martos A , López‐Pérez MJ & Montoya J (1996) In organello RNA synthesis system from mammalian liver and brain. Methods Enzymol 264, 50–57. 8965722 10.1016/s0076-6879(96)64008-7

[42] Čech P , Hoksza D & Svozil D (2015) MultiSETTER: web server for multiple RNA structure comparison. BMC Bioinformatics 16, 253. 26264783 10.1186/s12859-015-0696-8 PMC4531852

[43] Chomyn A (1996) In vivo labeling and analysis of human mitochondrial translation products. Methods Enzymol 264, 197–211. 8965693 10.1016/s0076-6879(96)64020-8

